# Comparative transcriptome profiling and morphology provide insights into endocarp cleaving of apricot cultivar (*Prunus armeniaca* L.)

**DOI:** 10.1186/s12870-017-1023-5

**Published:** 2017-04-11

**Authors:** Xiao Zhang, Lijie Zhang, Qiuping Zhang, Jiayu Xu, Weisheng Liu, Wenxuan Dong

**Affiliations:** 1 College of Horticulture, Shenyang Agricultural University, Shenyang, 110866 China; 2 Liaoning Institute of Pomology, Yingkou, 115009 China; 3 College of Forestry, Shenyang Agricultural University, Shenyang, 110866 China

**Keywords:** Apricot candidate genes, Comparative transcriptomes, Endocarp cleaving, Transcription factors

## Abstract

**Background:**

A complete and hardened endocarp is a typical trait of drupe fruits. However, the ‘Liehe’ (LE) apricot cultivar has a thin, soft, cleavable endocarp that represents 60.39% and 63.76% of the thickness and lignin content, respectively, of the ‘Jinxihong’ (JG) apricot (with normal hardened-endocarp). To understand the molecular mechanisms behind the LE apricot phenotype, comparative transcriptomes of *Prunus armeniaca* L. were sequenced using Illumina HiSeq™ 2500.

**Results:**

In this study, we identified 63,170 unigenes including 15,469 genes >1000 bp and 25,356 genes with Gene Function annotation. Pathway enrichment and expression patterns were used to characterize differentially expression genes. The DEGs encoding key enzymes involved in phenylpropanoid biosynthesis were significantly down-regulated in LE apricot. For example, CAD gene expression levels, encoding cinnamyl alcohol dehydrogenase, were only 1.3%, 0.7%, 0.2% and 2.7% in LE apricot compared with JG cultivar at 15, 21, 30, 49 days after full bloom (DAFB). Furthermore, transcription factors regulating secondary wall and lignin biosynthesis were identified. Especially for *SECONDARY WALL THICKENING PROMOTING FACTOR 1* (*NST 1*), its expression levels in LE apricot were merely 2.8% and 9.3% compared with JG cultivar at 15 and 21 DAFB, respectively.

**Conclusions:**

Our comparative transcriptome analysis was used to understand the molecular mechanisms underlie the endocarp-cleaving phenotype in LE apricot. This new apricot genomic resource and the candidate genes provide a useful reference for further investigating the lignification during development of apricot endocarp. Transcription factors such as NST1 may regulate genes involved in phenylpropanoid pathway and affect development and lignification of the endocarp.

**Electronic supplementary material:**

The online version of this article (doi:10.1186/s12870-017-1023-5) contains supplementary material, which is available to authorized users.

## Background

Apricot (*P. armeniaca* L.) is a typical drupe of the family Rosaceae with eight pairs of chromosomes (2 *n* = 16) [1]. Cultivated apricots are widely cultivated around the world (Asia 59.9%, Europe 21.6%, Oceania 0.4%, Africa 15.8%, and Americas 2.3%. FAO, 2013–2014) and apricot production has a relatively high economic value. Apricot fruit has rich nutritional value, including dietary fiber, organic acids, vitamin C, carotene, and trace elements [2]. Furthermore, the kernel is a natural plant protein resource, which used as medicine and food [3].

The pericarp develops from the ovary, and the innermost layer is the endocarp. The hardened endocarp has a vital role in seed protection and dispersal in some important economic fruits, such as peach, apricot, plum, almond, cherry, mango, olive, and coffee [4]. In plant evolution, the function of the heavily lignified endocarp is to ensure secure environment for seed development [5, 6]. Endocarp hardening is a significant trait of mature drupe fruits. It is caused by the secondary wall formation and lignin deposition [6]. Biochemical analysis has found that the endocarps of olive and peach contained much more lignin than poplar stem [7], suggesting that a relatively extreme degree of secondary wall formation occurs in fruit endocarp tissues. Lignin is an aromatic polymer that is widely found in the secondary walls of plants, as well as most enzymes and regulatory steps in the lignin biosynthetic pathway (phenylpropanoid pathway) have been identified [8]. Endocarp lignification in *Arabidopsis* has been adequately studied in relation to dehiscence, and even the transcriptional regulatory network has also been examined [9]. For drupe fruits, Ryugo [10] observed the regulation of lignin biosynthesis and accumulation in peach stones in the early 1960s. Lignification in peach endocarp is a highly coordinated process, which has been shown by subsequent developmental studies [11]. Recently, a transcriptional network dominated by *NAC* and *MYB* genes was observed in a well-conserved regulatory pathway, which causes *Arabidopsis* dehiscence or peach endocarp formation [4], and plays an essential role in the secondary wall formation and lignification via stimulating the pathway. Furthermore, several MADs-box genes involved in the formation of fruit endocarp, including *SHP1, SHP2*, *STK,* and *FUL* were identified [12]. These TFs co-function with *IND*, *ALC* [13], and *RPL* [14] to stimulates endocarp differentiation.

**Fig. 1:**
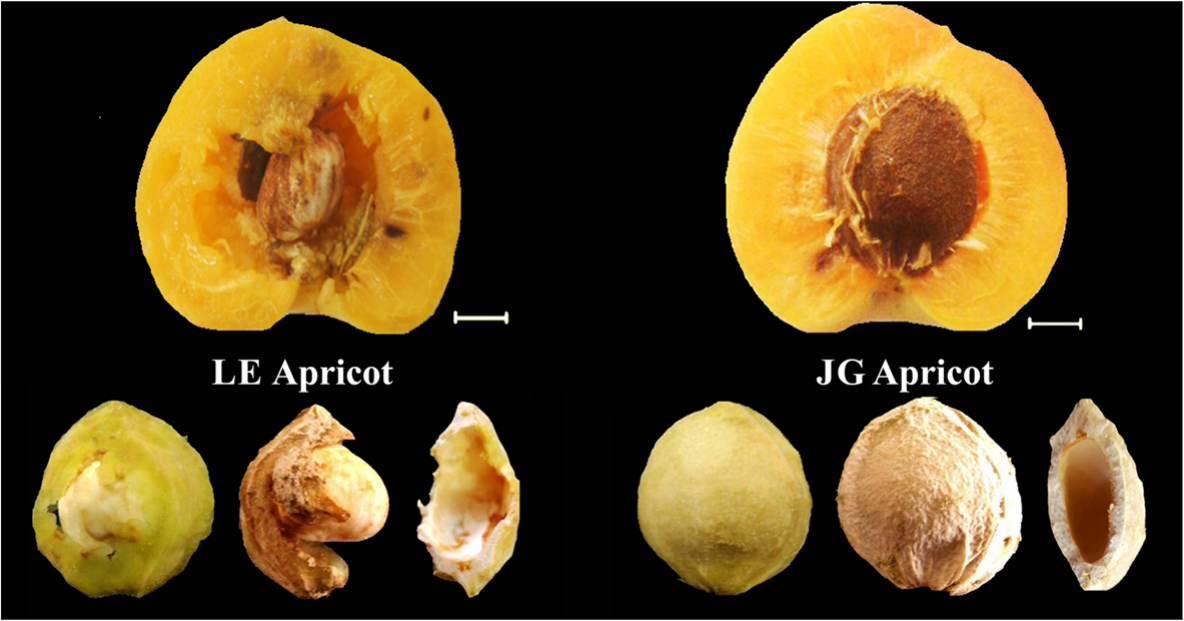
Morphology and structure of mature fruit and endocarp of LE and JG apricot in this study. *Left*: LE apricot with thin, soft and cleavable endocarp; *Right*: JG apricot with thick, hard and complete endocarp. The scale in this figure was 5 mm

However, there is wide variation in the phenotypes of *Prunus* endocarps, such as thickness, hardness, and brittleness of almond endocarps. The endocarp of “split pit” peach does not seal along the suture, leaving the seed severely exposed to pests and disease [15]. Callahan [16] found a natural “stoneless” plum in a wild-type *P. domestica*, which had imperfectly developed endocarp resulting in a partially naked seed. China has a great wealth of germplasm genetic resources of apricot that have important breeding values [17]. ‘Liehe’ (LE) apricot is an extremely rare cultivar that originated in Linyuan City of Liaoning Province and was introduced into National Germplasm Repository for Plums and Apricots (Xiongyue, Liaoning, China) in the 1983 [18]. The endocarp of LE apricot is thin, soft, and cleavable, and some seed partially exposed (Fig. 1). Both flesh and kernel have a tasty flavor and aroma.

This study investigated the mechanisms of endocarp development and phenotype formation, using Illumina sequencing and expression pattern analysis of candidate genes in apricot fruits during the stages of endocarp development and lignification.

## Results

### Differences in endocarp development in LE and JG apricot

**Fig. 2:**
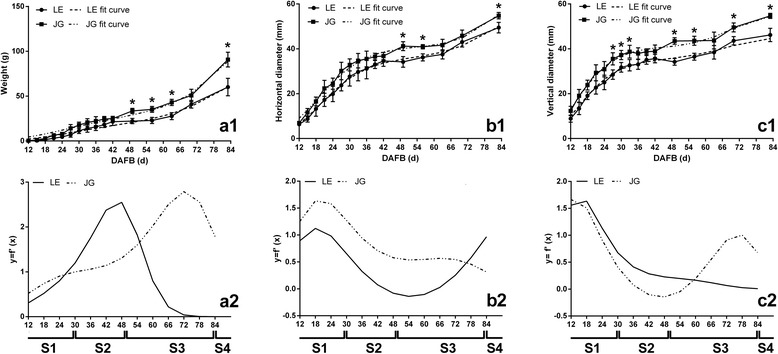
The changes of weight and growth curves of LE and JG apricot during fruit development stages. In **a1**, **b1**, **c1**, *points and solid lines* represent actual measured values, and the *dotted line* represent fit curves of equation. In **a2**, **b2**, **c2**, two different lines are fit curves of the first derivative of equations. Numbers under the x–axis indicate the days after full bloom. S1, first exponential growth stage; S2, slow–growth stage; S3, second exponential growth culminating in fruit ripening stage; S4, fruit ripening stage. *Error bars* indicate the standard deviation of ten biological replications. Label: ‘*’ means the significant differences at *P* < 0.05 by DMRT

The growth of LE apricot fruit was compared with JG apricot. The horizontal and vertical diameter of the two cultivars increased continuously during fruit development. Growth patterns were similar, with formal double-sigmoid growth curves (Fig. 2). The equation of two apricot cultivars and their first derivatives were also similar (Additional file 1: Table S1). In addition, the transcript level of *ACO1* and *PEPCK* divided the development of two apricots into same four stages (Additional file 2: Figure S1). Based on these patterns and fit equations, apricot fruit growth was assessed at four growth stages: S1, first exponential growth stage (before 30 DAFB); S2, slow-growth stage (30–49 DAFB); S3, second exponential growth culminating in fruit ripening stage (49–83 DAFB); S4, fruit ripening stage (after 83 DAFB). The changes in the growth pattern during fruit development in LE and JG apricot were almost the same.

**Fig. 3:**
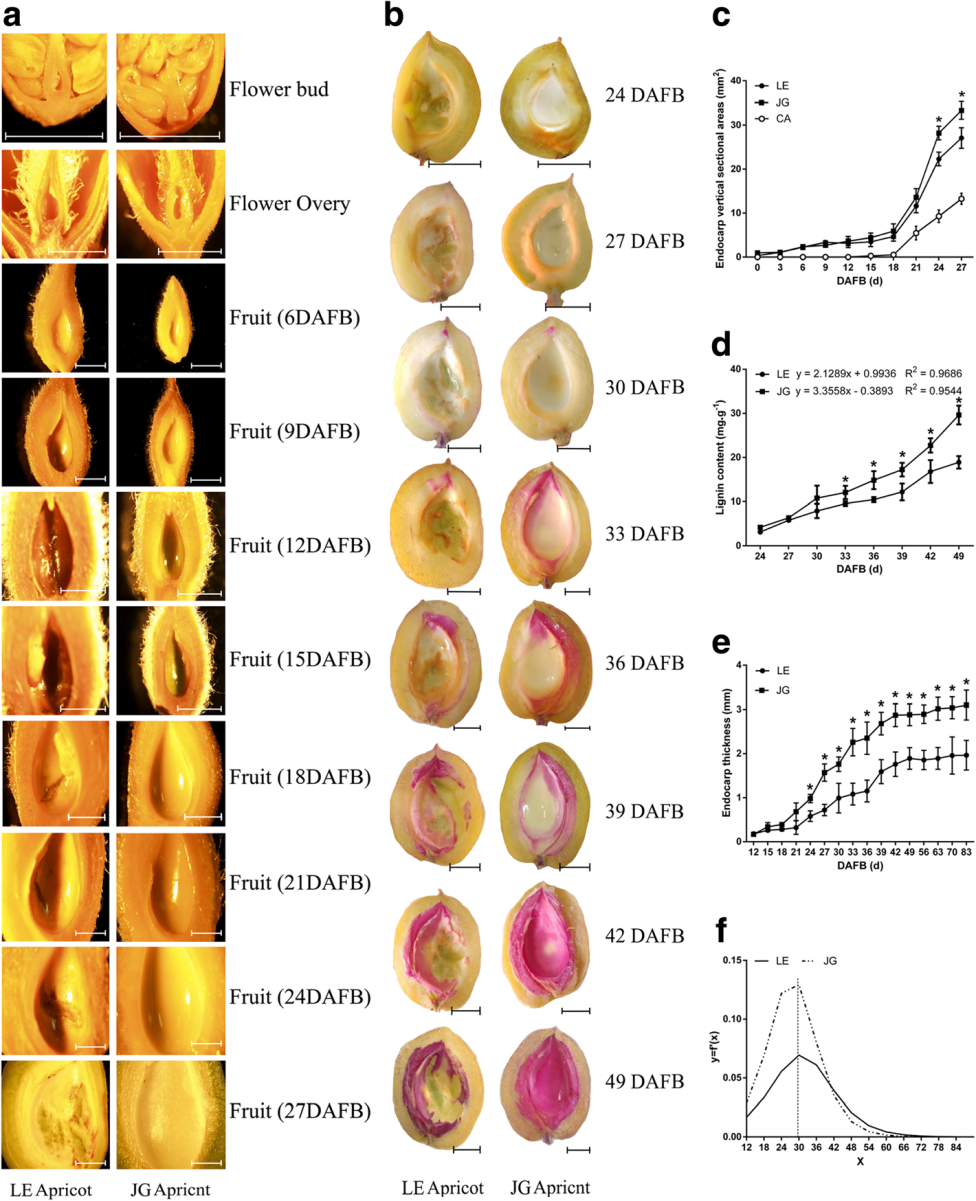
Observation of development and lignification of the endocarp in LE and JG apricot. **a** Microscopic observation of flowers and young fruits of two cultivars, the scale was 2 mm. **b** Observation of lignin deposition in two cultivars’ fruit, the scale was 5 mm. **c** Changes of endocarp vertical sectional areas in LE and JG apricot. ‘CA’ represent the cleaving areas of LE endocarp. **d** Changes of endocarp lignin content in LE and JG apricot. **e** Changes of endocarp thickness in LE and JG apricot. **f** The carve of first derivative of the endocarp thickness equation. Numbers under x–axis indicate the days after full bloom. *Error bars* indicate standard deviation of ten biological replications. Label: ‘*’ means the significant differences at *P* < 0.05 by DMRT

Flower buds, flowers and young fruits of LE and JG apricot were examined to investigate the features of the innermost layer of the apricot ovary (Fig. 3a). The innermost wall of the flower bud and flower ovary were smooth and normal, even in the early endocarp of young fruit with no obvious differences. However, at 15 DAFB, endocarp of LE apricot started to cleave, and these areas increased along with the progression in fruit development, which occurred in virtually each replicate sample of LE apricot. Endocarp thickness was significantly different after 30 DAFB subsequently (Fig. 3e, f), when cleaving areas of endocarp became more obvious in LE apricot (Fig. 3b, c).

The lignin deposition process was discernible from fruit transverse sections (at 24 DAFB) showing color reaction between the phloroglucinol and lignin (Fig. 3b). The lignin deposition process began at the tip of the endocarp and gradually completed over 25 days (24–49 DAFB). Interestingly, LE cultivar exhibited incomplete areas of endocarp that had little or no lignification (Fig. 3a, b). The thickness and lignin content were significantly lower in LE endocarp, which estimated by 60.4% and 63.3%, respectively; out of that in JG endocarp (Fig. 3d, e). Thus the differences in endocarp development and lignification were significant.

### Illumina sequencing and assembly

The LE endocarp cleaving occurred at 15 DAFB and rapidly increased at 21 DAFB (Fig. 3a). Thus, RNA from these two fruits stages of LE and JG apricot was used for RNA-seq, with two replicates per fruit, which generated 40,145,230,606 raw reads. After removing low-quality reads and trimming adapter sequences, 159,378,508 remained (Table 1). The assembly data were confirmed by an N50 value (1689 bp) and average length (868.72 bp). The number of transcripts (length ≥ 200 bp) was 152,146, constituting 99.99% of the total, with average lengths of 1579.75 bp (Table 1). Transcripts assembled 63,170 genes from LE and JG apricot. Among these genes, 15,469 had lengths of ≥1000 bp, constituting 24.49% (15,469/63,170) of the total (Table 2). Length distribution of all genes is shown in Table2.

**Table 1:** Summary of RNA–seq and de novo assembly of *P. armeniaca* L. unigenes

Sequence	Number
Total nucleotides	40,145,230,606
Numbers of clean reads	159,378,508
Numbers of 200–300 bp contigs	17,108,433 (99.71%)^a^
Mean length of contigs (bp)	39.07
N50 length of contigs (bp)	42
Numbers of ≥200 bp transcripts	152,146 (99.99%)^b^
Mean length of transcripts (bp)	1579.75
N50 length of transcripts (bp)	2598
Numbers of unigenes	63,170
Mean length of unigenes (bp)	868.72
N50 length of unigenes (bp)	1689

^a^The proportion of contigs (length 200–300 bp) to total contigs (17,158,454)

^b^The proportion of transcripts (length ≥ 200 bp) to total transcrips (152,146)

**Table 2:** Length distribution of *P. armeniaca* L. unigenes

All combination unigenes length (bp)	Total number	Percentage (%)
200–300	19,728	31.23
300–500	15,585	24.67
500–1000	12,388	19.61
1000–2000	8153	12.91
2000+	7316	11.58

### Functional annotation and identification of unigenes

Based on the sequence similarity, 25,356 genes were matched to the Japanese apricot and peach genome databases (Additional file 3: Table S2). All of these genes were aligned using BLASTx (E values ≤10^5^) searches against the NR, Swiss-Port, GO, COG, and KOG protein databases, and KEGG pathway databases. A total of 25,356 (40.14%) genes had more than one match, and 39.78% were annotated to the NR database (Table 3). Among the annotated genes in the NR database, 74.08% had an E-value ≤1.0 E^−5^ and showed very strong homology to the gene sequence in the database. The remaining 50.38% of genes had an E-value ranging from 1.0 E^−6^ to 1.0 E^−60^ (Additional file 4: Figure S2). We further analyzed the BLAST results in the NR database and investigated the best-hit species distribution, and the top two matched plant species were *P. mume* (63.67%) and *P. persica* (26.23%) (Additional file 4: Figure S2).

**Table 3:** Summary of assembled *P. armeniaca* L. unigenes

Database tpye	Number of unigenes length ≥ 300 bp	Number of unigenes length ≥ 1000 bp	The total number of annotated unigenes	Percentage (%)^a^
COG_Annotation	1405	5713	7724	12.23
GO_Annotation	3081	7856	12,347	19.55
KEGG_Annotation	1087	3214	4830	7.65
KOG_Annotation	3104	8290	12,735	20.16
Pfam_Annotation	3656	11,592	16,506	26.13
Swiss–prot_Annotation	4498	10,587	16,897	26.75
nr_Annotation^b^	7813	13,685	25,126	39.78
All_Annotation	7899	13,706	25,356	40.14

^a^Percentage means the proportion of 63,170 unigenes

^b^nr_Annotation means NCBI non–redundant sequence database

The functions of predicted genes were classified by GO analysis. A total of 16,506 genes annotated in the GO database were categorized into 57 functional groups, belonging to three main GO ontologies: biological processes, cellular components, and molecular functions (Additional file 5: Table S3; Additional file 6: Figure S3). ‘metabolic process’ (8408 genes, 50.93%), were dominant among the functional groups.

**Fig. 4:**
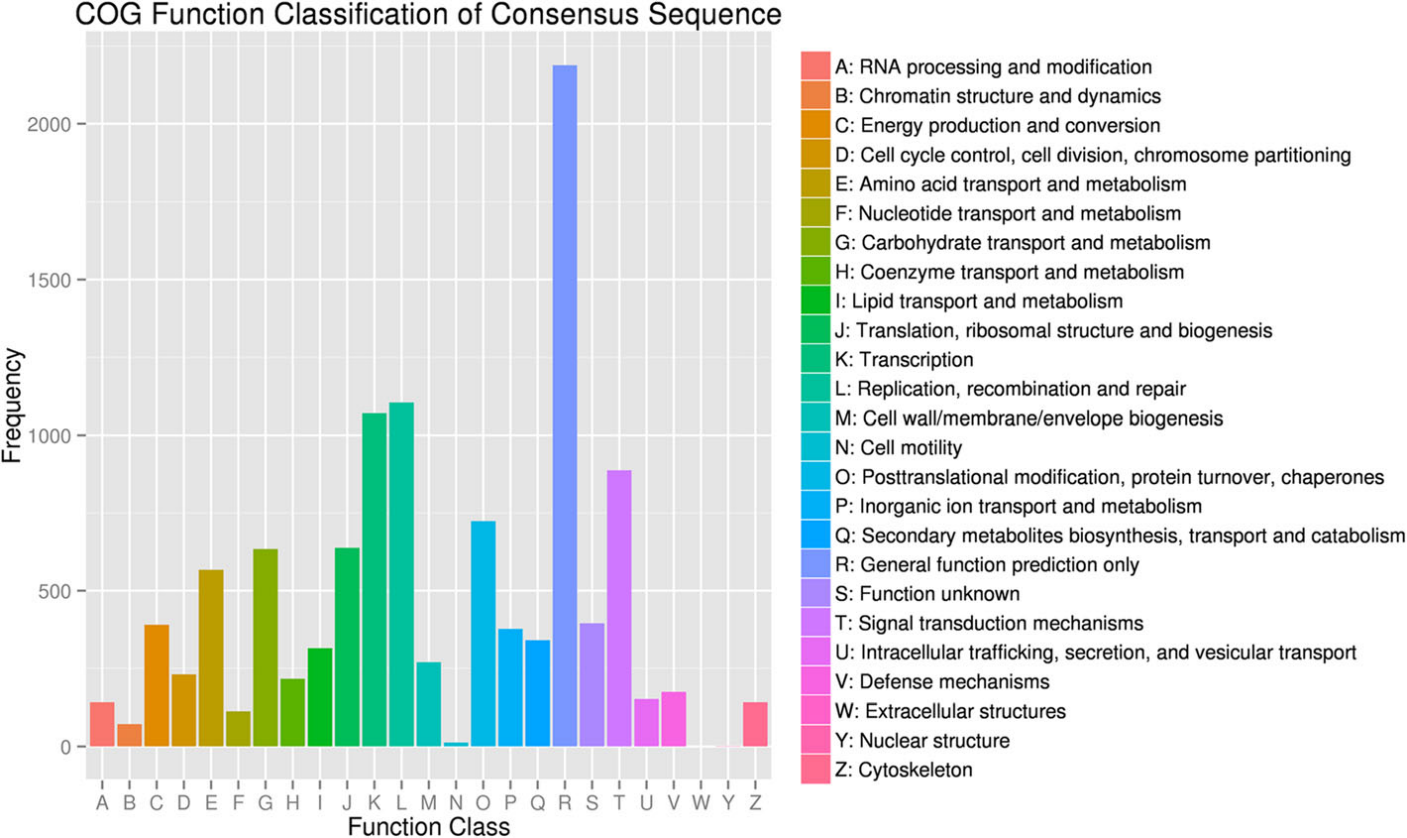
COG classification of assembled *P. armeniaca* L. unigenes

In addition, assembled genes were searched against the COG database to estimate the gene function (Fig. 4). In general, 7724 putative proteins were clustered into 25 functional categories. Among these categories, ‘general function prediction only’ (2188, 28.33%) accounted for the largest amount, followed by ‘replication, recombination and repair’ (14.29%) and ‘transcription’ (13.85%). In addition, 4.41% of assembled genes were assigned to secondary metabolites biosynthesis, transport, and catabolism, reflecting the large amount of secondary metabolites that were present in the apricot. The ‘nuclear structure’ (0.01%), ‘cell motility’ (0.17%), and ‘chromatin structure and dynamics’ (0.93%) accounted for the least amounts.

We used the KEGG pathway database to search the functional networks of biological interactions. In total, 4830 genes were identified in the KEGG database and were assigned to 118 KEGG pathways (Additional file 7: Table S4). The majority of genes was classified into pathways for ‘carbohydrate metabolism’ (905 genes), ‘translation’ (596 genes), ‘amino acid metabolism’ (581 genes), or ‘folding, sorting and degradation’ (471 genes). Biosynthesis of other secondary metabolites matched 192 genes.

**Fig. 5:**
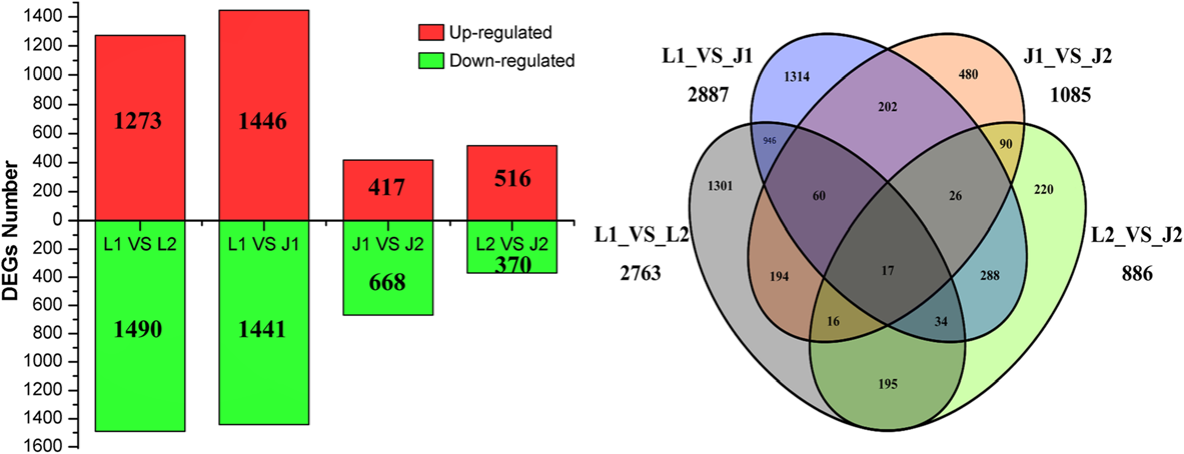
DEGs statistics and Venn diagrams between different cDNA libraries. *Red color* indicates up–regulated expression of DEGs; *Green color* indicates down–regulated expression of DEGs. L1 and J1: the DEGs were generated from LE relative to JG cultivar at 15 DAFB; L2 and J2: the DEGs of LE relative to JG cultivar at 21 DAFB. JG was always control sample

The expression patterns of the genes among LE1, LE2, JG1, and JG2 were calculated using the FPKM method. A total of 5385 DEGs were identified by comparing the four libraries in paired comparisons, as illustrated in Fig. 5. The most prominent library was LE1_vs_JG1. In each library, LE1_vs_LE2, LE1_vs_JG1, JG1_vs_JG2, and LE2_vs_JG2 had 2763, 2887, 1085, and 886 DEGs respectively. Four libraries had 17 common DEGs and 1301 DEGs in LE1_vs_LE2, as well as 1314 DEGs in LE1_vs_JG1, 480 DEGs in JG1_vs_JG2, and 220 DEGs in LE2_vs_JG2. These results indicated that early fruit development of apricot is a highly active process, and key genes that are related to endocarp development were significantly expressed.

### Transcript differences between LE and JG apricot

Endocarp cleaving and incomplete lignin deposition in the fruit of LE apricot were highly complex phenomena that caused by a series of biological processes, including many genes acting synergistically, collaborating in regulating various pathways. However, the phenylpropanoid pathway is undoubtedly one of the most important. Endocarp hardening occurs via secondary cell wall formation and lignification. In the phenylpropanoid pathway, *p*-coumaryl alcohol, sinapyl alcohol, and coniferyl alcohol are the end products that form the different types of lignin monomers [8]. From the KEGG enrichment analysis, phenylalanine metabolism, phenylpropanoid biosynthesis, and hormone signal transduction were the foremost pathways and contained the most number of DEGs in LE1_VS_JG1 (Additional file 7: Table S4; Additional file 8: Figure S4). Thirty-four DEGs associated with the phenylalanine pathway were differentially expressed.

**Fig. 6:**
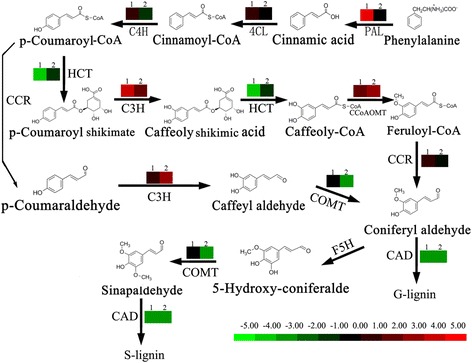
Expression of DEGs involved in phenylpropanoid pathway between LE and JG apricot. Expression pattern in this figure is from log_2_(FC). *Red color* indicates higher levels of gene expression in LE relative to JG cultivar; *Green color* indicates lower level of gene expression in LE relative to JG cultivar; *Black color* indicates gene expression had little difference between LE and JG cultivar. Label ‘1’ on the pattern indicates relative expression at 15 DAFB; ‘2’ indicates the relative expression at 21 DAFB

The expression level of genes which involved in phenylpropanoid pathway was down-regulated in LE compared with JG cultivar, in both the replicates and development stages (Table 4, Figs. 6, 8). These included genes encoding shikimate *O*-hydroxycinnamoyltransferase (HCT, unigene c42130.c0 and c26167.c0) [2.3.1.133], caffeic acid O-methyltransferase (COMT, unigene c43821.c0) [EC 2.1.1.6]. Among the seven annotated Peroxidase [EC 1.11.1.7] genes, two were down-regulated (unigene c10367.c0 and c36804.c0) in LE relative JG cultivar. Cinnamyl alcohol dehydrogenase (CAD, unigene c10104.c0) [EC 1.1.1.195] in particular, were involved in lignin biosynthesis and catalyzed the final step specific to the production of lignin monomers [19]. The expression level of *CAD* was always down-regulated in LE relative to JG cultivar during S1 stage. The fold change data of each selected candidate gene in the phenylpropanoid pathway are shown in Fig. 6, and detailed information is presented in Table 4.

**Table 4:** DEGs between LE and JG apricot that involved in phenylpropanoid pathway

	Fold change (log_2_ JG/LE)		
Unigene ID	LE1vsJG1	LE2vsJG2	Annotation
c36405.c0	3.72	−0.65	Phenylalanine ammonia–lyase 1 [*P. mume*]
c39178.c0	1.47	1.92	4–coumarate–CoA ligase [*A. thaliana*]
c13354.c0	2.45	––––	Cytochrome P450 CYP73A100 [*P. ginseng*]
c14455.c0	2.63	––––	Cytochrome P450 98A2 [*P. mume*]
c48482.c0	1.72	−2.29	Cytochrome P450 98A2 [*P. mume*]
c27758.c0	1.24	−0.32	Cinnamoyl–CoA reductase 1 [*A. thaliana*]
c15115.c0	7.29	7.96	Cinnamoyl–CoA reductase 1–like [*P. mume*]
c43821.c0	−0.21	−2.74	Caffeic acid 3–O–methyltransferase [*P. mume*]
c42130.c0	−2.77	−0.18	Shikimate O–hydroxycinnamoyltransferase [*A. thaliana*]
c26167.c0	−3.74	−2.10	Shikimate O–hydroxycinnamoyltransferase [*A. thaliana*]
c24524.c0	2.73	−1.86	Caffeoyl–CoA O–methyltransferase 1 [*A. thaliana*]
c10104.c0	−4.56	−2.10	Cinnamyl alcohol dehydrogenase [*A. thaliana*]
c9752.c0	−5.52	−5.11	Cinnamyl alcohol dehydrogenase [*A. thaliana*]
c10367.c0	−3.93	−1.31	Peroxidase 72 [*A. thaliana*]
c32572.c0	3.59	––––	Peroxidase 29 [*A. thaliana*]
c34865.c0	1.77	0.53	Peroxidase 12 [*A. thaliana*]
c36804.c0	−1.39	−4.52	Peroxidase 42 [*A. thaliana*]
c41877.c0	2.55	−1.74	Peroxidase 4 [*V. vinifera*]
c35483.c0	2.36	−0.71	Peroxidase 17 [*A. thaliana*]
c35595.c0	1.63	−0.41	Peroxidase 51 [*A. thaliana*]
c45746.c0	–1.64	0.09	Aspartate aminotransferase [*D. carota*]
c41467.c2	1.24	−1.09	aminotransferase TAT2 [*A. thaliana*]
c10544.c0	2.28	−1.71	Cytochrome P450 98A3 [*A. thaliana*]

Several TFs were identified that mediated the endocarp development, including SHP and STK. ALC and IND promoted endocarp differentiation and negative regulation was achieved by FUL and RPL. Meanwhile, NST1, NST3, and several MYB-box genes are associated with secondary wall formation and lignin biosynthesis. By the RNA-seq, the majority of these TFs and genes were identified and showed in Fig. 8. The expression of *SHP*, *FUL*, and *MYB32* were up-regulated in LE relative to JG cultivar. However, *STK*, *MYB46–1*, *MYB46–2*, and *NST1* were down-regulated significantly in LE compared with JG cultivar (Table 5, Fig. 8).

**Table 5:** DEGs between LE and JG apricot that involved in secondary wall biosynthesis

	Fold change (log_2_ JG/LE)		
Unigene ID	LE1 VS JG1	LE2 VS JG2	Annotation
c33854.c0	−0.30	−0.33	Agamous–like MADS–box protein AGL11 [*A. thaliana*]
c37786.c0	0.49	0.27	Agamous–like MADS–box protein AGL1 [*A. thaliana*]
c31419.c1	0.37	0.51	Agamous–like MADS–box protein AGL8 [*A. thaliana*]
c32638.c0	1.30	−0.01	BEL1–like homeodomain protein 9 [*A. thaliana*]
c10986.c0	−1.93	−1.32	NAC domain–containing protein 43 [*A. thaliana*]
c23553.c0	−1.69	−0.07	NAC domain–containing protein 7 [*A. thaliana*]
c45336.c0	−3.56	−3.62	Gibberellin 3–beta–dioxygenase 1 [*P. sativum*]
c34462.c0	–0.03	1.27	Transcription factor MYB46 [*A. thaliana*]
c36170.c1	–0.55	0.29	Transcription factor MYB46 [*A. thaliana*]
c37609.c0	1.98	1.99	Transcription factor MYB32 [*P. mume*]

**Fig. 7:**
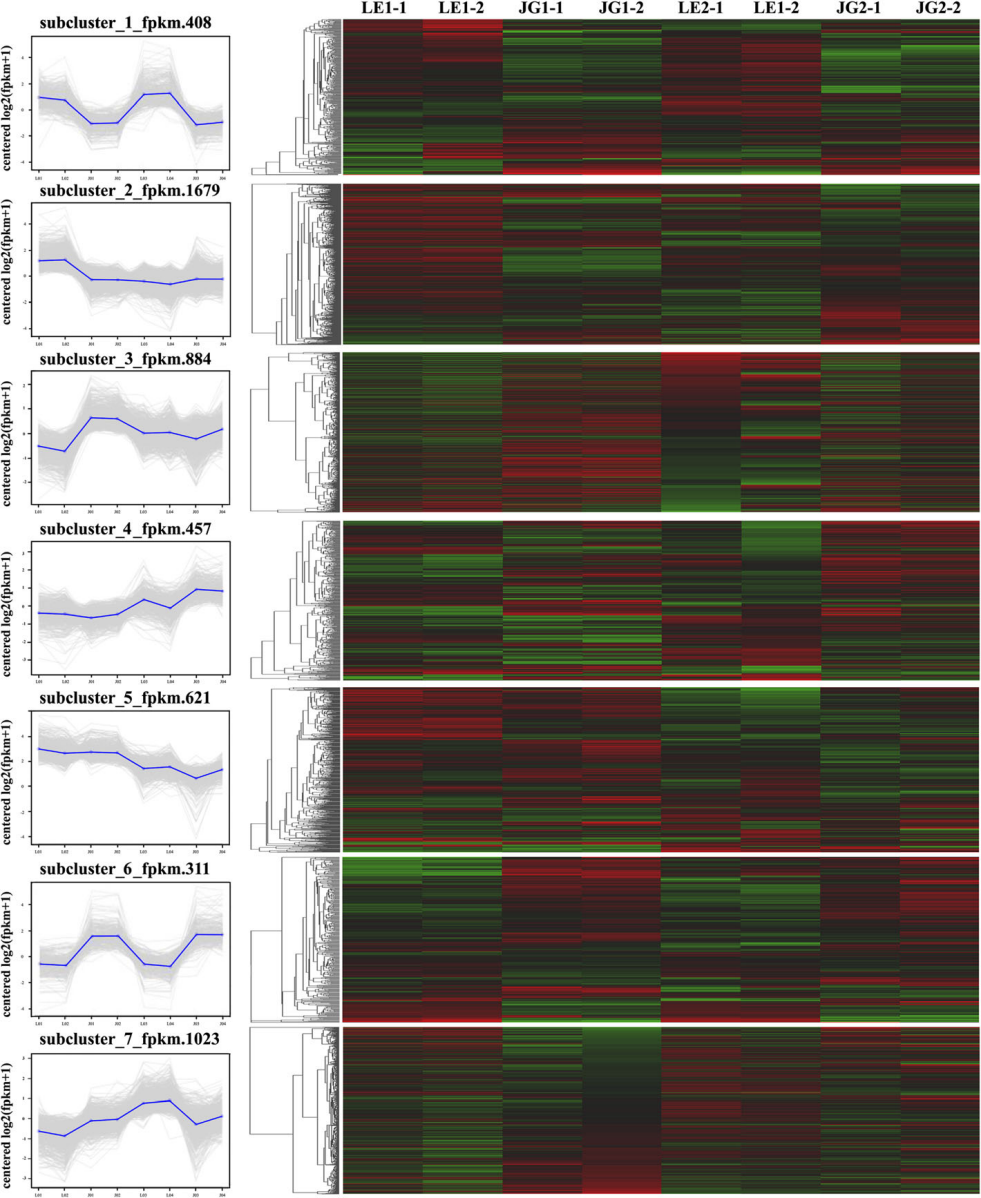
Common expression analysis based on DEGs expression patterns. *Left*: Numbers under the x-axis indicated different sample, numbers under the y-axis indicated the log_2_(fpkm + 1). *Right*: *Red color* represents higher log_2_(fpkm + 1) data of genes; *Green color* represents lower log_2_(fpkm + 1) data of genes. *Black color* represents log_2_(fpkm + 1) = 0. LE1–1 and LE1–2 indicate two biological replications of LE at 15 DAFB; LE2–1 and LE2–2 indicate two biological replications of LE at 21 DAFB; JG1–1 and JG1–2 indicate two biological replications of JG at 15 DAFB; JG2–1 and JG2–2 indicate biological replications of JG at 21 DAFB

We used common expression patterns to further analyze the DEGs between LE and JG apricot at 15 and 21 DAFB. Based on this method, 5383 DEGs were placed into seven clusters (Fig. 7; Additional file 9: Table S5). Most of the candidate DEGs was categorized in either Cluster 1 (408 genes) or Cluster 6 (311 genes). Compared with JG apricot, the expression of DEGs of LE apricot was up-regulated in Cluster 1, and some DEGs were present as *CHS1*, *F3H*, *CSLG3*, *CSLA9*, *KATAM*, *ARR5*, and *ARR16* (Additional file 9: Table S5). Conversely, the expression of DEGs such as *DFR* and *XTH2* in LE apricot was down-regulated in Cluster 6.

**Fig. 8:**
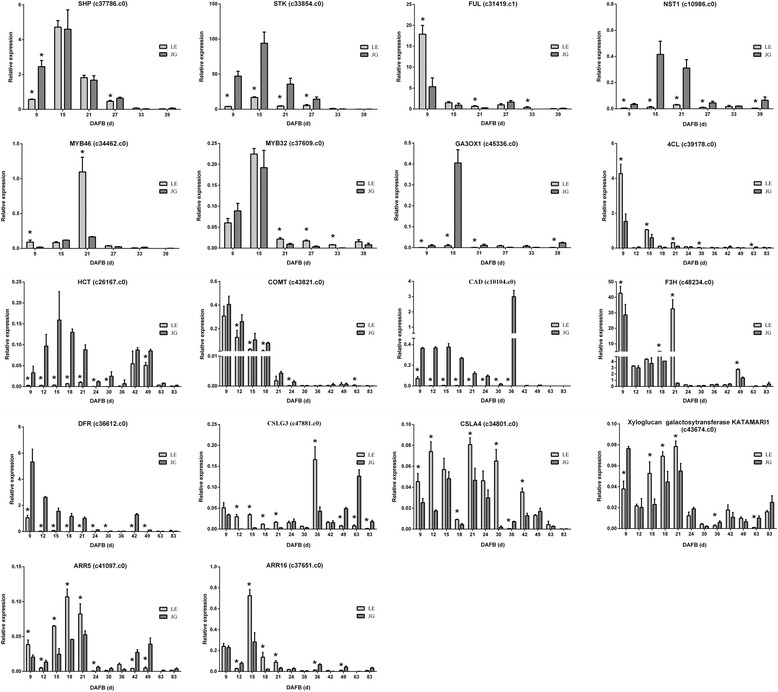
qPCR analyses of selected candidate genes involved in this study. *Light–colored bars* indicate LE apricot; *dark–colored bars* indicate JG apricot. Numbers under the x–axis indicate the days after full bloom. Label: ‘*’ means the significant differences at *P* < 0.05 by DMRT

There was a linear correlation (*R* = 0.9188, *P* ≤ 0.0001) between RNA-seq data and qPCR in our study (Additional file 10: Figure S5). TFs (*STK*, *SHP*, *FUL*, *NST1*, *MYB46*, and *MYB32*) and key candidate genes could regulate fruit endocarp growth, development, and lignification (Fig. 8). These genes involved in the biosynthesis of plant hormones (*GA3ox1*, *ARR5*, and *ARR16*), phenylpropanoid pathway (*4CL*, *HTC*, *COMT,* and *CAD*), flavonoid biosynthesis (*F3H* and *DFR*), and cellulose-related pathway (*CSLG3*, *CSLA9*, and *KATAM*). *STK*, *NST1*, *GA3ox1*, *HCT, COMT* and *CAD* were down-regulated and *MYB32* was up-regulated in LE apricot, compared with JG, in RNA-seq data and gene expression, respectively. Furthermore, Pearson’s correlation analysis indicated that there was a significant association between *CAD* expression and endocarp thickness in LE apricot at the 0.05 level. *HCT* expression and lignin content also showed the same result (Additional file 11: Table S6). The special endocarp development and lignification in LE were caused by the effects of several TFs and genes involved in phenylpropanoid pathway.

## Discussion

The hardened endocarp has a vital role in seed protection and dispersal in some important economic fruits, such as peach, apricot, plum, almond, cherry, mango, olive, and coffee [4]. Endocarp hardening is a significant trait of fruit matures of any types of drupes, which caused by the secondary wall formation and lignin deposition [6]. Phenylpropanoid biosynthesis played a crucial role in endocarp lignification in both LE and JG apricot. Sequence analysis of transcriptome revealed a series of differentially expressed genes involved in the phenylpropanoid pathway, such as *4CL*, *HCT*, *COMT* and *CAD*. Knockout of *4CL* in *Arabidopsis* had no significant effect on either lignin content or monomeric composition [20]. However, RNAi silencing of *HCL* in *Arabidopsis* and *Radiata pine* reduced lignin content and changed the monomeric composition [21, 22]. In *COMT* antisense *Leucaena leucocephala,* the lignin content was reduced to 72% by decreasing 60% of OMT activity [23]. *CAD1* made a significant contribution to the synthesis of coniferyl alcohol, and down-regulated *CAD1* in wild-type tobacco has a moderate impact on G unit content of the non-condensed lignin fraction [24]. CAD activities were drastically reduced in null mutants of *Arabidopsis* (*AtCAD-D* and *AtCAD-C*), and affected sinapyl alcohol dehydrogenase activity in these mutants. *AtCAD-D* had an significant influence on lignin content and proportion of conventional S lignin [25]. In LE cultivar, expression of *4CL* were up-regulated, yet expressions of *HCL* and *COMT* were down-regulated, which relative to JG cultivar (Figs. 6, 8). In particular, *CAD* expressed down-regulated in LE apricot compared with JG, in both RNA-seq data and relative gene expression (Figs. 6, 8). These results indicated that thickness and incomplete endocarp are unlikely to result from mutation of one specific phenylpropanoid pathway gene. In fact, expression levels of *CAD* and *HCT* had significant correlation with endocarp thickness and lignin content in LE apricot (Additional file 11: Table S6). These genes or TFs may be responsible for the defects in endocarp development and lignification in LE apricot.

*SHP*, *STK* and *NST1* were specifically expressed in endocarp of peach. In exocarp and mesocarp, the negative regulator *FUL* exhibited a high expression level. However, the expression of *IND* and *ALC* was insignificant [4]. In *Arabidopsis stk shp1 shp2* triple mutants, the integuments were changed into carpel-like structures leading to complete sterility [26]. Over-expression of *FUL* caused no lignin deposition in valve tissues in *Arabidopsis* [27]. Furthermore, in tomato, over-expression of *FUL2* lead to a thinner pericarp, and reduced stem cell layer [28]. In a split pit resistant variety of peach, *SHP* expression was low, however in the sensitive variety, *FUL* expression was significantly elevated [15]. Our analysis found that *STK*, *SHP*, and *FUL* were discovered in DEGs, but *IND* and *ALC* were not. Expression of *SHP* and *FUL* had significant different between LE and JG at 9 DAFB, while *STK* remained down-regulated during the S1 stage significantly. RNA-seq data and qPCR analysis reflected that *SHP*, *STK*, and *FUL* were highly expressed and essential for endocarp development (Table 5; Fig. 8). IND directly activates GA3ox1, which is an indispensable enzyme catalyzing the last step of GAs biosynthesis in the separation layer of *Arabidopsis*. IND induces GAs accumulation to degrade DELLA protein, resulting in release ALC [29]. In *atga3ox1 atga3ox2* double mutants of *Arabidopsis*, synthesis of cellulose, hemicelluloses, and lignin were suppressed obviously [30]. NST1 and NST3 (SND1) have been proven as master switches that regulate the secondary wall biosynthesis and lignification in *Arabidopsis* [31], *Medicago* [32], and Poplar [33]. In the transcriptional network, the downstream transcription factor MYBs is activated by NST1 and SND1, and multiple genes are involved in secondary wall biosynthesis [34]. In *nst1* mutants, valve margins were obvious in the absence of the secondary wall, meanwhile in *nst1 nst3* double mutants, only vascular vessels conserved secondary wall formation [35]. The *SND1*, and *VND1–5*, *VND6–7* were not detected as DEGs in this study. The expression levels of *VND4* were always lower in LE relative to JG cultivar at 15 and 21 days after full bloom (Additional file 3: Table S2). *NST1*, the domain of that regulates biosynthesis of secondary wall, lignin and xylanase always had low expression levels in LE fruit (Table 5; Fig. 8). This might be one of the main cause of cleaving and thinning of endocarp in LE apricot. Hence, *NST1* was regarded as an essential candidate gene in the development and phenylpropanoid biosynthesis in endocarp of LE apricot. MYB46 is also a decisive master switch and *AtMYB46* was reported to be a direct target of ANAC012/SND1/NST3 [36], which adjusted secondary cell wall biosynthesis [37]. Dominant repression or over-expression of MYB46 has a considerable effect on secondary wall thickening of fibers and vessels and biosynthesis of lignin and cellulose [36]. We identified two differentially expressed *MYB46* (Table 5). In addition, MYB46 could activate the expression of MYB4, MYB7, and MYB32 [38], and the MYB32 protein sequence was highly similar to that of MYB4 [39]. *AtMYB4* regulates the expression of *C4H*, so that *AtMYB32* could negatively regulate several genes implicated in phenylpropanoid biosynthesis [40]. Trans-activation assays and transgenic studies also show that MYB32 appears to be a negative regulator of SND1 expression [32]. Interestingly, *MYB32* was extraordinary up-regulated in LE, and expression of *C4H* down-regulated (Fig. 8) only in LE fruit. MYB32 of apricot might also play an important part in negative regulating the lignin biosynthesis in the secondary wall.

Common expression pattern analysis provided a new understanding of the expression and function of DEGs, and combines pathways with multiple candidate genes, which were related to the flavonoid pathway and cell function (Fig. 7; Additional file 9: Table S5). *CHS1* and *F3H* expressed higher level in LE than JG, which may cause by considerable down-expression of *CAD*. However, *DFR* had a down-regulated expression. Among 408 genes in Cluster 1, *CSLG3*, and related genes, *CSLA9* [41] and *KATAM* had significant higher level expression in LE during stage S1 and S2 relative to JG cultivar. Furthermore, *ARR5* and *ARR16* regulators appeared to act as negative regulators of Cytokinin signaling [42], and showed significantly up-regulated expression in LE apricot.

## Conclusions

Our results implied that cleaving of endocarp in LE apricot started at 15 DAFB, and this area increased during fruit development. The thickness and lignin content of the mature LE endocarp was only 60.39% and 63.25%, respectively, compared with JG endocarp (Fig. 3). RNA-Seq to sequencing and de novo assembly of the fruit transcriptomes of two cultivars of *P. armeniaca* (L.) showed discrepancies in development and lignification of the endocarp and explained the cleaving of endocarp in LE apricot. The DEGs and qPCR analysis data (Fig. 8) identified differentially expression genes involved in TFs (STK, SHP, FUL, NST1, MYB46, MYB32) and phenylpropanoid, flavonoid and hormone pathways (*4CL*, *HCT*, *COMT*, *CAD*, *CHS1*, *F3H*, *DFR*, *GA3ox1*, *ARR5*, and *ARR16*), consistent with endocarp phenotype and lignin content. Our results indicated that TFs especially *NST1*, may regulate genes of the phenylpropanoid pathway. Besides, low expression level of *NST1* may inhibit the endocarp development and lignification of LE apricot.

## Methods

### Plant materials

‘Liehe’ (LE) apricot, synonym as ‘Luoren’ apricot, and ‘Jinxihong’ (JG) apricot are local cultivars originated respectively in Linyuan City and Jinxi County of Liaoning Province and were collected into National Germplasm Repository (N40°10′1.18″, E122°09′39.41″) for Plums and Apricots at Xiongyue, Liaoning, China in 1983. LE and JG, with the accession number XC0347 and XC0015 based on the Chinese National Key Project “Exploration, Collection, Conservation of Plum and Apricot Germplasm Resources” funded by the Agricultural Ministry of China. The identification of LE and JG cultivar was done by the Liaoning Institute of Pomology [43, 44]. During the 2015 season, the fruits of LE and JG were picked in National Germplasm Repository for Plums and Apricots from 6 DAFB (50% of flowers had opened) including all the developmental stages of fruit, with the permission of the curator, Dr. Weisheng LIU, of National Germplasm Repository for Plums and Apricots and used in this study. The seeds were separated from the fruit which were picked. The stages were characterized by weight and shape (horizontal and vertical diameter). Fruits were sliced, frozen in liquid N_2_, and stored at −80 °C for RNA extraction.

### Measurement of fruit growth and endocarp lignification

Fresh fruit diameter were measured (horizontal and vertical diameter) by digital Vernier caliper (0–150 mm ± 0.02 mm), and weighted using electronic scales (300 g/0.01 g). The fitting for each equation were used the Pearl-Reed logistic [45, 46] and normal logistic equation [47] as references. The first derivative of equation was calculated and drew by MATLAB 8.5 (Math Works, US). The expression levels of transcripts encoding ACO1 and PEPCK were analysis used the same method [48]. Flower buds, flowers and young fruits were observed using an Olympus SZX7 microscope to examine the cleaving of the endocarp. Developing endocarp areas were calculated using Olympus cellSens software. Fruit samples for lignin deposition observation were collected once every 3 days from 15 to 43 DAFB. Observation and lignin content tests were conducted using Alba’s method [49]. The measurements for each index were repeated three times in 10 samples. Means and analysis of variance (ANOVA) were separated using Duncan’s Multiple Range Test (DMRT) in SPSS 19.0 (IBM, US).

### cDNA library preparation and Illumina sequencing

Total RNAs were extracted from fruit samples without kernels using Gambino’s method [50]. The RNA samples were examined with an Agilent 2100 Bioanalyzer (US). cDNA library preparation and sequencing of fruits at 15 DAFB (LE1, JG1) and 21 DAFB (LE2, JG2) with two replicates per each cultivar, were conducted by the Biomarker Technology Company (Beijing, China). The cDNA library used high-throughput sequencing (RNA-seq) with the Illumina HiSeq™ 2500. Reads length of sequences was PE125.

### Sequence assembly and functional annotation

A large number of raw reads was produced using Sequencing by Synthesis (SBS) from Illumina HiSeq™ 2500. The Trinity method [51] was used for de novo assembly of Illumina reads of the two apricot cultivars. Clean reads were mapped to the genome of *Prunus mume* and *Prunus persica* using TopHat Software [52]. Genes were first aligned using BLASTx (E value <10^5^) to the NCBI non-redundant protein databases (NR) [53]. The alignments from the NR database were used blast2GO (https://www.blast2go.com/) to get GO annotation [54]. The number of DEGs which matched to three categories was counted, and GO ontology figure was drawn by Graph-R Project. The statistical method of GO enrichment was “right sided Fisher exact test”. The term was Core ontology (go.obo, http://purl.obolibrary.org/obo/go.obo). The main parameter of BLASTx is “blastx -task blastx-fast -num_descriptions 100 -num_alignments 100 -evalue 1e-5”. This parameter was used to blast databases. The annotation of genes were performed using the method were as follows: Swiss-Port protein databases [55], COG [56], KOG [57] KEGG [58]. Predicted amino acid sequences were aligned by hidden Markov models (HMMER, E value <10^10^) [59] to the Protein family (Pfam) [60] to annotate the genes. Coding sequence (CDS) of genes were predicted by TransDecoder Software (http://transdecoder.github.io).

### Differentially expression genes analysis

Gene expression levels were analyzed using fragments per kilobase of the transcript per million mapped reads (FPKM) method [61]. DESeq Software [13] was used to identify DEGs in pair-wise comparisons, and the results of all statistical tests were revised to account for multiple testing with the Benjamini–Hochberg false discovery rate (FDR <0.01). Sequences were determined to be significantly differentially expressed at a *P* value (<0.01), and Fold change (FC) >2. Common expression pattern analysis using BMKCloud (https://www.biocloud.net/) was applied twice to serial samples. Euclidean distance was used in the Distance method and K-means for hierarchical clustering. Hierarchical clustering was conducted using Spotfire DecisionSite 8.1 (Spotfire Inc., http://spotfire.tibco.com/).

### Quantitative RT-PCR analysis

A total of 500 ng of RNAs was used to synthesize cDNA using PrimeScript™RT Kit (Cat. RR047A, TaKaRa, Japan). The cDNA was diluted five times, and then used as a template. The reaction solution contained SYBR® PremixExTaq™ II (Tli RNaseH Plus) (Cat. RR820A, TaKaRa, Japan) and was conducted in an ABI 7500 Real Time PCR Detection System (Applied Biosystems, US). Quantitative primers for validation of DEGs are listed in Additional file 12: Table S7. The relative expression levels of the selected genes, normalized to peach *ACT* [62] and *P. mume ACT7* (unigene, c48143.c0), were calculated using the 2^-ΔCt^ method. All reactions were performed with three biological replicates. Three technical replicates were in each biological replicate. The analysis of variance (ANOVA) was based on Duncan’s Multiple Range Test (DMRT) in SPSS 19.0 (IBM, US).

## Additional files

Additional file 1: Table S1. Growth curve equation and its first derivative of *P. armeniaca* L. (XLS 33 kb)

Additional file 2: Figure S1. Transcription levels of genes marking different phonological phases of apricots. (TIFF 484 kb)

Additional file 3: Table S2. Summary of DEGs and annotation. DEGs were generated for comparison between LE and JG apricot and JG was control sample. (XLS 2860 kb)

Additional file 4: Figure S2. E-value and NR distribution of assembled *P. armeniaca* L. unigenes. (TIFF 891 kb)

Additional file 5: Table S3. Summary of GO enrichment analyses of assembled *P. armeniaca* L. unigenes. DEGs were generated for comparison between LE and JG apricot and JG was control sample. (XLS 46 kb)

Additional file 6: Figure S3. GO classification of assembled *P. armeniaca* L. unigenes and DEGs. The results were summarized in three main GO categories: cellular component, molecular function, and biological process. ‘metabolic process’ (50.93%), ‘cellular process’ (42.41%), ‘single-organism process’ (36.33%) ‘binding’ (37.30%), ‘catalytic activity’ (40.42%), ‘cell part’ (31.31%), and “cell” (31.17%) were dominant among the functional groups. DEGs were generated for comparison of LE and JG apricot and JG was control sample. The right y-axis indicated the number of assembled unigenes and DEGs. (TIFF 9700 kb)

Additional file 7: Table S4. KEGG pathway analysis of *P. armeniaca* L. assembled unigenes. (XLS 185 kb)

Additional file 8: Figure S4. KEGG enrichment analyses of DEGs between LE and JG apricot at 15 DAFB. Phenylalanine metabolism (Q value =0.032), Phenylalanine biosynthesis (Q value =0.055). Red color represents higher expression levels of genes in LE relative to JG apricot; Green color represents lower expression levels of genes in LE relative to JG apricot. (TIFF 3033 kb)

Additional file 9: Table S5. Annotation of DEGs between LE and JG apricot in Cluster1 and Cluster6. (XLS 264 kb)

Additional file 10: Figure S5. Correlation analysis of fold change data of RNA–seq with that from qPCR. 18 genes were selected for this analysis. (TIFF 183 kb)

Additional file 11: Table S6. Correlation between lignin related phenotypic measurements and DEGs expression data. (XLS 44 kb)

Additional file 12: Table S7. Primers used to perform qPCR of selected candidate genes. (XLS 35 kb)

## Abbreviations

ACO1: 1-aminocyclopropane-1-carboxylic acid oxidase 1
ALC: Basic helix-loop-helix genes *ALCATRAZ*
ARR16: Two-component response regulator ARR16
ARR5: Two-component response regulator ARR5
CAD: Cinnamyl alcohol dehydrogenase
CDS: Coding sequence
CHS1: Chalcone synthase 1
COG: Clusters of orthologous groups databases
CSLA9: Glucomannan 4-beta-mannosyltransferase
CSLG3: Cellulose synthase 3
DAFB: Days after full bloom
DEGs: Differentially expression genes
DFR: Dihydroflavonol-4-reductase
DMRT: Duncan’s Multiple Range Test
F3H: Flavanone 3-hydroxylase
FPKM: Fragments per kilobase of the transcript per million mapped reads
FUL: MADS-Box genes *FRUITFUL*
GA3ox1: Gibberellin 3-beta-dioxygenase 1
GO: Gene ontology databases
HCT: Shikimate *O*-hydroxycinnamoyltransferase
IND: Basic helix-loop-helix genes *INDEHISCENT*
KATAM: Xyloglucan galactosyltransferase KATAMARI1 homolog
KEGG: Kyoto encyclopedia of genes and genomes pathway databases
KOG: euKaryotic orthologous groups databases
NR: NCBI non-redundant protein databases
NST3: *SECONDARY WALL THICKENING PROMOTING FACTOR*
PEPCK: Phosphoenolpyruvate carboxykinase
qPCR: Quantitative real-time PCR
*REPLUMLESS*: NST1
RPL: BEL1-like homeodomain gene
SHP: MADS-box genes *SHATTERPROOF*
STK: MADS-box genes *SEEDSTIC*
TF: Transcription factor
VND1–7: Protein VASCULAR RELATED NAC-DOMAIN 1–7
XTH2: Xyloglucan endotransglucosylase/hydrolase 2
