# Supplementary figures and images for: Comparative transcriptome profiling and morphology provide insights into endocarp cleaving of apricot cultivar (Prunus armeniaca L.)
Source: BMC Plant Biol. 2017 Apr 11;17:72. doi: 10.1186/s12870-017-1023-5 (PMC5387262; doi:10.1186/s12870-017-1023-5)

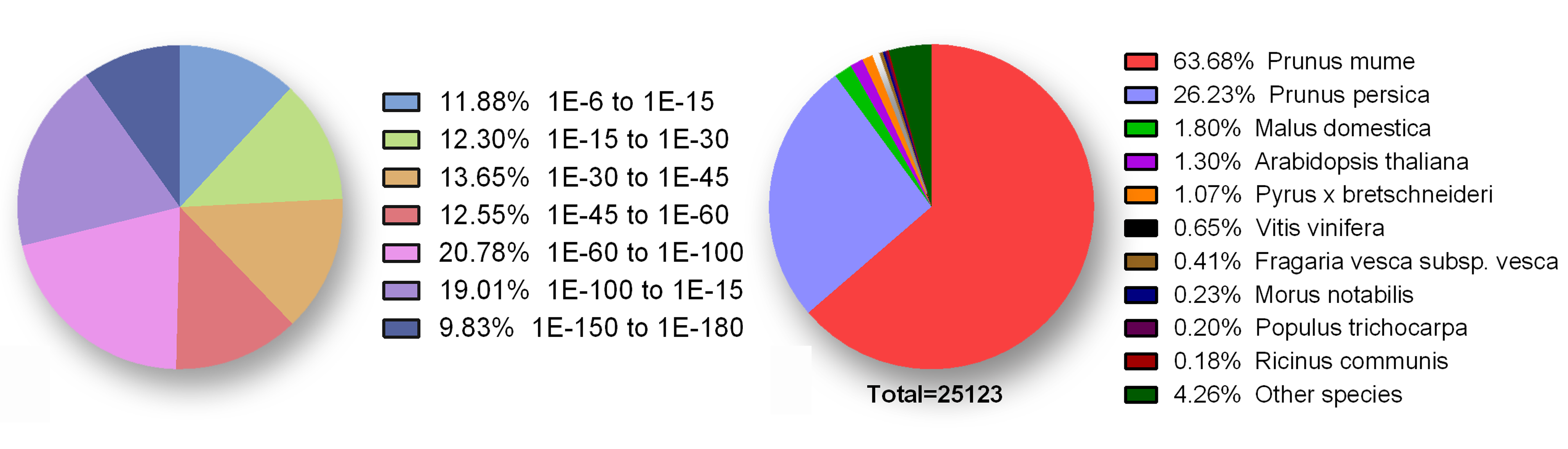

Supplement: Supplementary file 4 — E-value and NR distribution of assembled P. armeniaca L. unigenes. (TIFF 891 kb) [file 12870_2017_1023_MOESM4_ESM.tif]

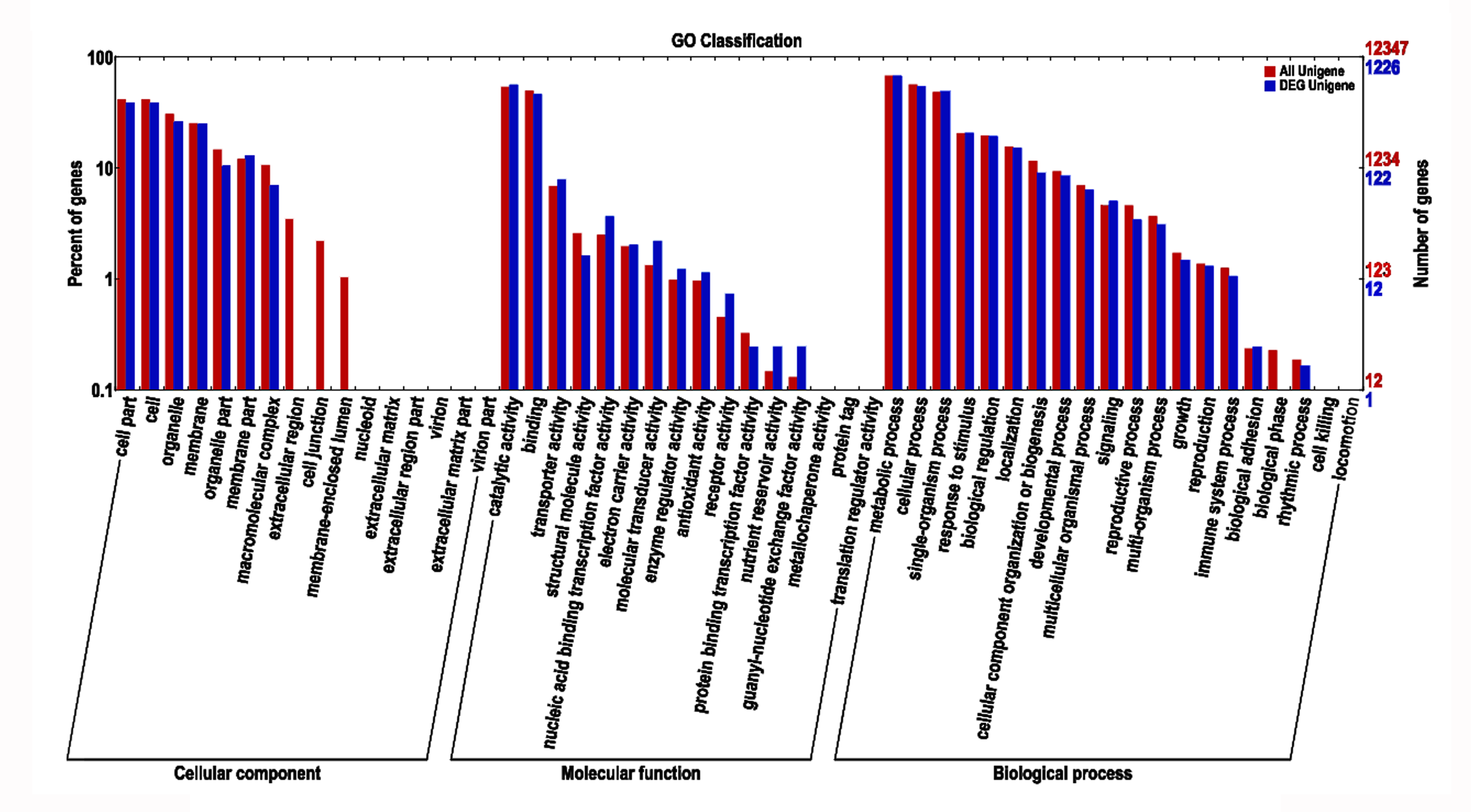

Supplement: Supplementary file 6 — GO classification of assembled P. armeniaca L. unigenes and DEGs. The results were summarized in three main GO categories: cellular component, molecular function, and biological process. ‘metabolic process’ (50.93%), ‘cellular process’ (42.41%), ‘single-organism process’ (36.33%) ‘binding’ (37.30%), ‘catalytic activity’ (40.42%), ‘cell part’ (31.31%), and “cell” (31.17%) were dominant among the functional groups. DEGs were generated for comparison of LE and JG apricot and JG was control sample. The right y-axis indicated the number of assembled unigenes and DEGs. (TIFF 9700 kb) [file 12870_2017_1023_MOESM6_ESM.tif]

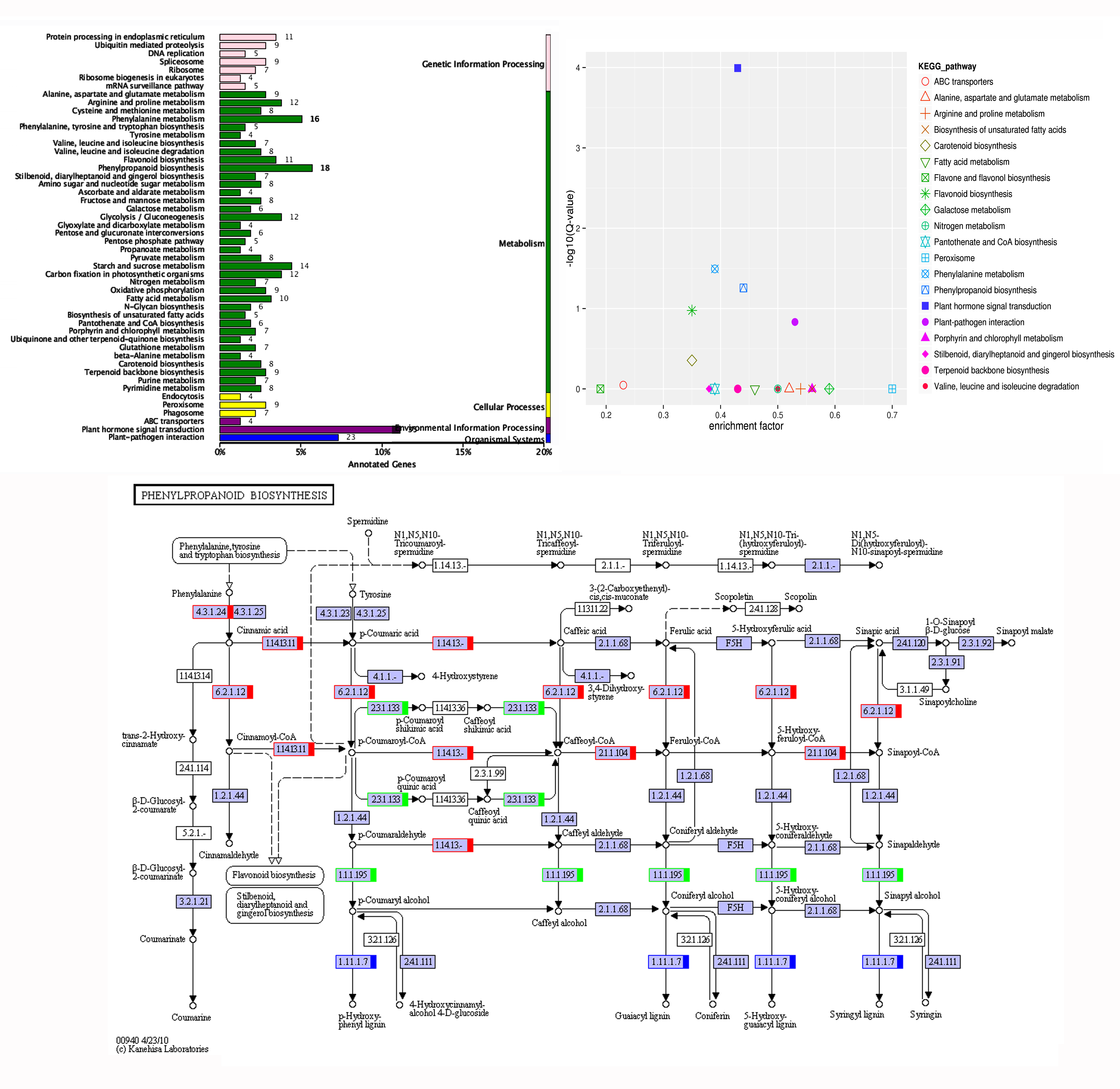

Supplement: Supplementary file 8 — KEGG enrichment analyses of DEGs between LE and JG apricot at 15 DAFB. Phenylalanine metabolism (Q value =0.032), Phenylalanine biosynthesis (Q value =0.055). Red color represents higher expression levels of genes in LE relative to JG apricot; Green color represents lower expression levels of genes in LE relative to JG apricot. (TIFF 3033 kb) [file 12870_2017_1023_MOESM8_ESM.tif]
